# NPTXR as a novel therapeutic target: efficacy of antibody-drug conjugates in preclinical tumor models

**DOI:** 10.1186/s12935-026-04438-5

**Published:** 2026-08-07

**Authors:** Mykola Lyndin, Michel Janicot, Justus Müller, Peter Schiemann, Gunther Wennemuth

**Affiliations:** 1 Department of Anatomy, University of Duisburg-Essen, Medical Faculty, Hufelandstrasse 55, 45147 Essen, Germany; 2 Ymmunobio AG, Gartengasse 12, Riehen, 4125 Switzerland

**Keywords:** Antibody–drug conjugate, Neuronal pentraxin receptor, NPTXR, oncology

## Abstract

**Background:**

The neuronal pentraxin receptor (NPTXR) is mainly expressed in the cytoplasm of a subset of neuronal cells in the cerebral cortex. While NPTXR may be involved in mediating uptake of synaptic material during synapse remodeling or synaptic clustering of AMPA glutamate receptors at a subset of excitatory synapses during embryonic development, *NPTXR* expression has recently been shown to be elevated in tissues from patients with gastric cancer.

**Methods:**

NPTXR protein expression was evaluated in human cancer and healthy tissue samples. We then generated antibody–drug conjugates based on YB-800 (YB-800_ADCs_), a fully humanized monoclonal antibody selectively targeting NPTXR. The cross-reactivity of YB-800 and YB-800_ADCs_ were assessed, as well as their tumor inhibitory effects in experimental tumor models.

**Results:**

NPTXR protein was highly and consistently expressed in human tissue samples from multiple cancer types but only minimally expressed, if at all, in healthy tissues. Treatment with YB-800_ADCs_ inhibited tumor cell proliferation/survival in engineered HEK293 cells overexpressing the human NPTXR. YB-800_ADCs_ were also found to have pronounced anti-tumor effects in mice bearing NPTXR-expressing HEK293 tumors at well-tolerated doses. Initial anti-tumor findings were further confirmed using a naturally occurring NPTXR-expressing human bladder PDX model.

**Conclusions:**

NPTXR is an oncofetal protein that is highly expressed in multiple human cancers but minimally expressed in healthy tissues. We have established NPTXR as a new tumor marker and have developed antibody–drug conjugates using YB-800, a novel, first-in-class, humanized monoclonal antibody targeting the human NPTXR. Initial results demonstrate that YB-800_ADCs_ have pronounced anti-tumor effects in vivo in NPTXR-expressing cancer cells. Continued evaluation of YB-800_ADCs_ is warranted.

**Supplementary Information:**

The online version contains supplementary material available at 10.1186/s12935-026-04438-5.

## Background

The neuronal pentraxin receptor (NPTXR) is a type-II transmembrane protein that is mainly expressed in the hippocampus and cerebral cortex and may play a critical role in synapse formation and synaptic organization during early brain development [1–6]. Besides its role in synaptic transmission, NPTXR does not appear to have any major physiological functions; *NPTXR* knockout mice do not exhibit any developmental, reproductive, metabolic, or motor function abnormalities, and treating mice for up to 6 weeks with anti-NPTXR monoclonal antibodies is not associated with any adverse effects [7].

NPTXR has recently been shown to play a role in the proliferation, migration, adhesion, and apoptosis of gastric cancer cells in vitro and in vivo [7, 8]. Recent work has also shown that *NPTXR* expression is higher in tissues from gastric cancer patients with distant metastasis *versus* those without, while *NPTXR* overexpression is associated with disease progression and worsened survival in patients with gastric cancer [7]. *NPTXR* silencing has also been shown to promote caspase-mediated apoptosis and to attenuate gastric cancer cell proliferation in vitro.

While NPTXR protein expression has already been suggested as a novel biomarker of disease recurrence in patients with gastric cancer [7], we hypothesize that NPTXR may be useful as a novel tumor marker in other cancers. Furthermore, there have also been encouraging reports that indicate NPTXR may be a viable therapeutic target. In a mouse xenograft model of peritoneal human gastric metastasis, for instance, a monoclonal antibody against NPTXR has been shown to inhibit tumor formation and to enhance the cytotoxicity of chemotherapy [7].

In this study, we aimed to evaluate expression of NPTXR in human cancer samples and healthy tissues to determine its potential as a novel selective tumor marker. We then aimed to generate and evaluate a first-in-class monoclonal antibody that binds to NPTXR with high affinity. We used this antibody to construct antibody–drug conjugates using a third-generation linker technology allowing cleavable and non-cleavable attachment of multiple mono and dual chemotherapeutic payloads. This optimized approach, combining a new tumor target and advanced antibody–drug conjugate technology, was used to optimize a novel treatment approach for cancer patients with tumors expressing NPTXR.

## Methods

### Expression of NPTXR in human cancer and healthy tissues

Expression of NPTXR in cancerous and healthy tissues was assessed using tissue microarrays. Unstained slides with healthy human and cancer tissues were purchased from a commercial vendor (TissueArray LLC, Derwood, USA, https://www.tissuearray.com). Unstained cancer tissue slides were also purchased from an additional commercial vendor (ProVitro AG, Berlin, Germany, https://provitro.com). All human samples were de-identified in compliance with vendor policies.

Tissue microarrays included samples from bladder cancer, cervical cancer, squamous non-small-cell lung cancer, pancreatic cancer, breast cancer, colon cancer, endometrial cancer, bile duct cancer, ovarian cancer, esophageal cancer, kidney cancer, thyroid cancer, lymphoma, rhabdomyosarcoma, and prostate cancer, and relevant samples from healthy tissue. All samples used in tissue microarrays were received from certified hospitals that guaranteed that all human tissue samples were collected with informed consent from the donors and their relatives, were excised by licensed medical doctors, and were diagnosed and identified by at least two different evaluators; donors remained anonymous, and all tissue was identified by ID code only.

NPTXR protein expression was evaluated by immunofluorescence (IF) using the anti-NPTXR antibody YB-800 (see below) diluted 1:1000 in 1.0% BSA/PBS as the primary antibody and biotinylated goat anti-human diluted 1:500 in 1.0% BSA/PBS as secondary antibody (Invitrogen, Thermo Fisher Scientific Cat. No. 31770). Cy3-streptavidin (Jackson ImmunoResearch, Cat. No. 016-160-084) was used to visualize biotinylated secondary antibodies bound to NPTXR, while DAPI (Serva, Cat. No. 18860) was used to stain cell nuclei. Both reagents were diluted 1:200 in 1.0% BSA/PBS and applied simultaneously as a single staining cocktail. Full details can be found in the Supplementary Methods.

IF results were evaluated semi-quantitatively by assessing the intensity of staining and the percentage of positively stained cells in 3 to 5 representative non-overlapping regions of interest (ROIs), avoiding edges, folds, necrosis, crush artifacts, and hemorrhages. Only viable invasive tumor cells were scored, with a minimum count of at least 500 cells across ROIs. Staining intensity was determined using either a fluorescence microscope or validated digital image viewer and graded on a 0 to 3 scale calibrated to positive controls (background was confirmed with a negative control using a nonspecific immunoserum of the host animal instead of the primary antibody). A staining grade of 0 indicated no detectable specific staining, 1 indicated weak staining that was barely above background, 2 indicated moderate staining, and 3 indicated strong staining with a robust or saturating signal. The percentage of cells in each ROI demonstrating each intensity grade was estimated. Percentages were averaged across ROIs and H-scores were calculated using the following formula: H-score = (1 × percentage of cells with low-intensity staining) + (2 × percentage of cells with medium-intensity staining) + (3 × percentage of cells with high-intensity staining). Therefore, H-scores may range from 0 to 300 (low intensity: 0–100, medium intensity: 101–200; high intensity: 201–300), and an H-score threshold of ≥ 150 was considered to represent sufficient target expression to allow for sufficient antibody–drug conjugate binding and internalization to achieve therapeutic efficacy. Further details of H-score assessment can be found in the Supplementary Methods.

### Quantification of NPTXR signal in tumor, metastatic, and healthy tissue

Quantification of the Cy3-positive area (corresponding to NPTXR signal) was performed using ImageJ software (NIH, Bethesda, MD, USA). Multichannel images were split into individual channels, and the red channel (Cy3) was analyzed. A threshold was applied to generate a binary mask, with signal pixels set to white and background to black. Measurements were performed within manually defined ROIs of identical size across all images. For statistical analysis, the Cy3-positive area was normalized to the total ROI area.

### Evaluating NPTXR expression in mice

Since the mouse and human NPTXR protein sequences are highly conserved, expression of NPTXR in mouse tissues was assessed using YB-800. Tissue microarrays were evaluated by IF. Tissue microarrays were derived from Kunming strain mice and included samples from liver, kidney, lung, skeletal muscle, cerebrum, cardiac muscle, stomach and spleen; samples were taken from 20 individual animals. All animal procedures were approved by Rincon Bioscience LLC’s Institutional Animal Care and Use Committee (IACUC protocol number: RIN-004, approved November 15, 2024) and conducted in accordance with institutional guidelines and applicable national regulations. Expression of NPTXR was assessed using the same protocol as for human tissue microarrays.

## Monoclonal YB-800 antibody production

### Antibody humanization

Based on the variable heavy and light chain antibody sequence of the mouse antibody as provided by Prof. Kanda, Nagoya University, Japan, a human antibody germline for the artificial intelligence (AI)-assisted complementarity-determining region (CDR) grafting humanization approach was selected based on the known productivity, abundance, and potential degree of germline identity.

Humanized antibody variants were created by CDR-grafting (using a wildtype human IgG1 isotype with a functional fragment crystallizable (Fc) region) and structural analysis onto the selected germline. The resulting humanized variable chains were synthesized and cloned into mammalian human expression vectors. All individual heavy and light chain variable sequences as well as the mouse sequence were shuffled with each other resulting in different antibodies with different degrees of humanization. Heavy-chain and light-chain antibody genes were used for transient transfection of mammalian cells. After one-step purification of produced antibodies by protein A affinity chromatography and buffer exchange to PBS (pH7.4, no additives), all variants were compared to the parental antibody in terms of functionality, specificity, stability and biophysical characteristics to select the best antibody candidate with the highest degree of germline identity (Supplementary Table S1 and Supplementary Figure S1). Produced and purified IgG antibodies were measured by UV/VIS spectrometry (280 nm). Antibodies with low production yield compared to the mouse chimeric antibody were excluded from lead candidate selection. The analysis was continued by reducing SDS-PAGE, and antibodies showed the expected heavy (approximately 50 kDa) and light chain (25 kDa) antibody pattern. An antigen binding ELISA was also performed; the biotinylated human NPTXR protein and biotinylated mouse peptide of NPTXR were immobilized via streptavidin to an ELISA plate and, after blocking and washing, the antibodies were tested in a titration series starting from 10 µg/ml and detected using a secondary anti human Fc antibody (Sigma A0170). The top three candidates based on production yield, binding and especially degree of humanness were further characterized by receptor inhibition testing and stability testing. Briefly, YB-800-induced NPTXR inhibition was evaluated using ELISA to determine blocking of NPTX2 binding to NPTXR using 1 µg/ml of ligand (or bovine serum albumin as control), 0.42 µg/ml of biotinylated receptor, and titration of antibody (concentrations ranged from 0.0001 µg/ml to 10 µg/ml) in PBS with Tween. Stability testing was also performed by analytical high-performance liquid chromatography size exclusion chromatography (HPLC-SEC) before and after stress induction. The antibody was either incubated at 45 °C for 48 h or for 24 h in a citrate buffer at pH 3.0, and both stress-treated samples were compared to the non-treated control and the chimeric antibody. The chimeric antibody showed strong tailing by analytical HPLC-SEC indicating a mixed antibody fraction; after pH treatment the tailing was significantly stronger and a secondary low molecular weight peak occurred, indicating fragmentation or degradation of the antibody. All three lead humanized variants showed improved stability, but the highest stability was observed for the fully humanized YB-800 (anti-NPTxR_VH1_VK1) antibody. The polyreactivity of the three lead candidates was also compared to the parental antibody by ELISA. After immobilization of four biomolecules (DNA, lysozyme, cell lysate and LPS), the antibodies were tested for binding to these molecules. No major differences compared to the parental antibody were seen and all antibodies showed minimal polyreactivity in the range of the therapeutic control antibody tested in parallel (palivizumab).

Based on all data, the anti-NPTxR_VH1_VK1 antibody with fully human frameworks was selected as the final fully humanized YB-800 antibody, since it showed improved stability, the highest degree of humanness, and no major drawbacks.

### Larger-scale production of YB-800

The fully humanized YB-800 was produced transiently in CHO cells and purified by protein A affinity chromatography and preparative SEC as well as sterile filtration (0.22 μm) for antibody–drug conjugation and further testing. The purified 99% monomeric antibody was tested for endotoxin and found negative with an endotoxin level < 0.02 EU/mg making it suitable for all cell assays. Binding was evaluated by ELISA and EC_50_ calculation as well as affinity measurement by Octet biolayer interferometry (Sartorius, Göttingen, Germany). Briefly, affinity measurement was carried out by immobilizing 3 µg/mL of IgG antibody to a protein A sensor and a titration of NPTXR protein was performed using a seven-point concentration series from 500 nM to 0.5 nM, with subsequent dissociation in assay blocking buffer. Data were analyzed by global fitting; three concentrations of YB-800 were selected (15.8 nM, 1.58 nM, and 5 nM), (Supplementary Figure S2). ELISA was performed by immobilization of NPTXR to a plate and titration of antibody and detection using an anti-human Fc secondary antibody. The antibody showed a K_D_ (representing binding affinity to human NPTXR) of 3.6 × 10^− 10^ M (360 pM) and an EC_50_ (representing avidity) of approximately 133 pM. Stability was further tested by differential scanning fluorimetry to determine heat stability; two major unfolding events were observed, both in the range of two therapeutic control antibodies tested in parallel (trastuzumab and palivizumab). The first unfolding occurred at 68.6 °C and the second at 81.4 °C, while macroscopic aggregation was seen from 70.2 °C onwards and unfolding started at 62.8 °C, indicating high thermal stability of the humanized YB-800 antibody (Supplementary Figure S2C).

### Antibody–drug conjugate generation and antibody testing

Two YB-800–based antibody–drug conjugates were generated with distinct payload compositions using a proprietary platform from T-E Meds (Taipei, Taiwan) as illustrated in Supplementary Figure S3. YB-800_MMAF_ consisted of YB-800 coupled via non-cleavable linkers to eight molecules of monomethyl auristatin F (MMAF), a potent antimitotic agent. In contrast, YB-800_MMAF−EXT_ contained four non-cleavably linked MMAF molecules and four cleavably linked exatecan (EXT) molecules, a DNA topoisomerase I inhibitor.

Conjugation was achieved by introducing azido groups into the YB-800 glycan structure and attaching proprietary two-arm drug bundles through strain-promoted azide–alkyne cycloaddition. Both antibody–drug conjugates were prepared at high efficiency and purified by hydrophobic interaction chromatography, yielding homogeneous conjugates with the expected drug–antibody ratios. Structural integrity was confirmed by SDS-PAGE and chromatographic profiling. Affinity measurements using a protein A biosensor demonstrated that YB-800_MMAF_ and YB-800_MMAF−EXT_ retained high binding activity toward recombinant NPTXR, with kinetics comparable to the parental antibody (Supplementary Figure S4). These findings indicated that glycan-directed conjugation enables stable incorporation of multiple cytotoxic payloads without compromising antigen recognition. Drug-to-antibody ratio analysis data and purity data for YB-800_MMAF_ and YB-800_MMAF−EXT_ are shown in Supplementary Figure S5.

## Cells and cell culture

### Generation of NPTXR-expressing human cancer cell line

Although strong and consistent binding of YB-800 was demonstrated in human tumor tissue microarrays in an extended range of tumor indications (Table 1), very limited, if any, inconsistent binding of the antibody was observed in classical commercially available cell lines (data not shown). We therefore set out to engineer *NPTXR*-expressing tumor cells to assess activity of YB-800_ADCs_ in cell-based assays and experimental tumor models in immune-compromised mice.

SV40 large T antigen-transformed human embryonic kidney HEK293T cells were initially purchased from ATCC (CRL-3216; Manassas, VA USA). Cells were cultured in RPMI 1640 (containing L-Glu) cell culture medium complemented with 10-mM Hepes buffer pH 7.4, and 10% fetal bovine serum at 37 °C in a humidified incubator with 5% CO_2_ atmosphere. HEK293T cells were engineered to stably express the human NPTXR gene, as indicated in the legend of Supplementary Figure S6.

Following the transfection procedure, polyclonal control HEKMZ3 (HEK293 transfected with no NPTXR-containing plasmid) and NPTXR-expressing (T1016) cells were selected for further analysis. HEKMZ3 and T1016 were maintained and cultured in RPMI 1640 (containing L-Glu) cell culture medium complemented with 10 mM Hepes buffer (pH 7.4), 10% fetal bovine serum, and 1 mg/mL G418 (for T1016) at 37 °C in a humidified incubator with 5% CO_2_ atmosphere.

### Testing of YB-800_ADCs_ in cell-based assays

The effects of parental YB-800 antibody, YB-800_MMAF_, and YB-800_MMAF−EXT_ were evaluated on HEKMZ3 and T1016 cell proliferation and survival using an Agilent xCELLigence Real-Time Cell Analysis SP instrument (Agilent Technologies, Santa Clara, CA, USA). The Agilent xCELLigence system measures electrical impedance across microelectrodes embedded in the bottom of specialized microtiter plates (E-Plates). As cells adhere to and proliferate on these electrodes, they alter the impedance, which is detected by the system and reported as a ‘cell index’ (CI) value, an arbitrary unit. Increasing CI values indicate more attached cells, reflecting cell proliferation and/or viability.

Parental YB-800 and YB-800_ADCs_ were evaluated in T1016 and HEKMZ3 (control) cells for time- and concentration-dependent cell proliferation and survival using the xCELLigence technology platform. Experimental conditions were validated with the use of increasing concentrations of cisplatin as an experimental positive control.

**Fig. 1:**
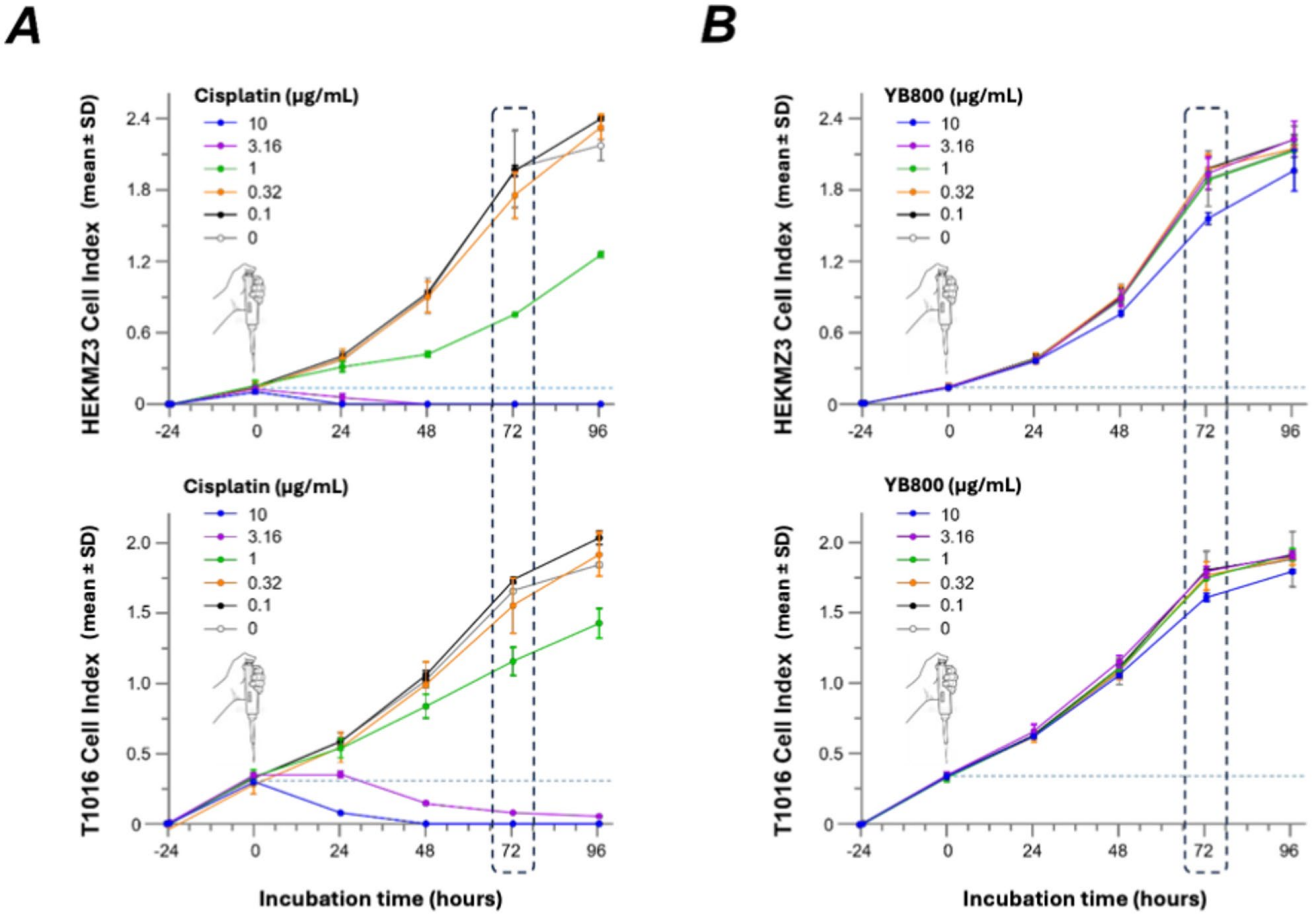
Time- and concentration-dependent effect of cisplatin or YB-800 on either control HEKMZ3 or T1016 cell proliferation/survival. HEKMZ3 (no *NPTXR* expressing cells) or T1016 engineered cells (transfected with human NPTXR-containing plasmid) were seeded on microtiter E-plates as indicated in the Methods section, and cells were allowed to adhere to plastic for 24 h at 37 °C in a humidified cell culture incubator in 5% CO_2_ atmosphere. Cells were then treated with the indicated concentrations (µg/mL) of (**A**) cisplatin or (**B**) parental YB-800 antibody and were monitored over a period of 96 h under similar cell culture conditions. Impedance was measured at indicated timepoints using the Agilent xCELLigence Real-Time Cell Analysis (RTCA) SP instrument, and reported as a cell index (CI) value. Specifically, the CI measurements occurred at cell seeding, 4 and 24 h after cell seeding (before compound addition), and at 24, 48, 72 and 96 h after compound addition. In this experiment, vehicles were sterile water (cisplatin) and PBS (YB-800). Data from 3 experimental replicates are shown

**Fig. 2:**
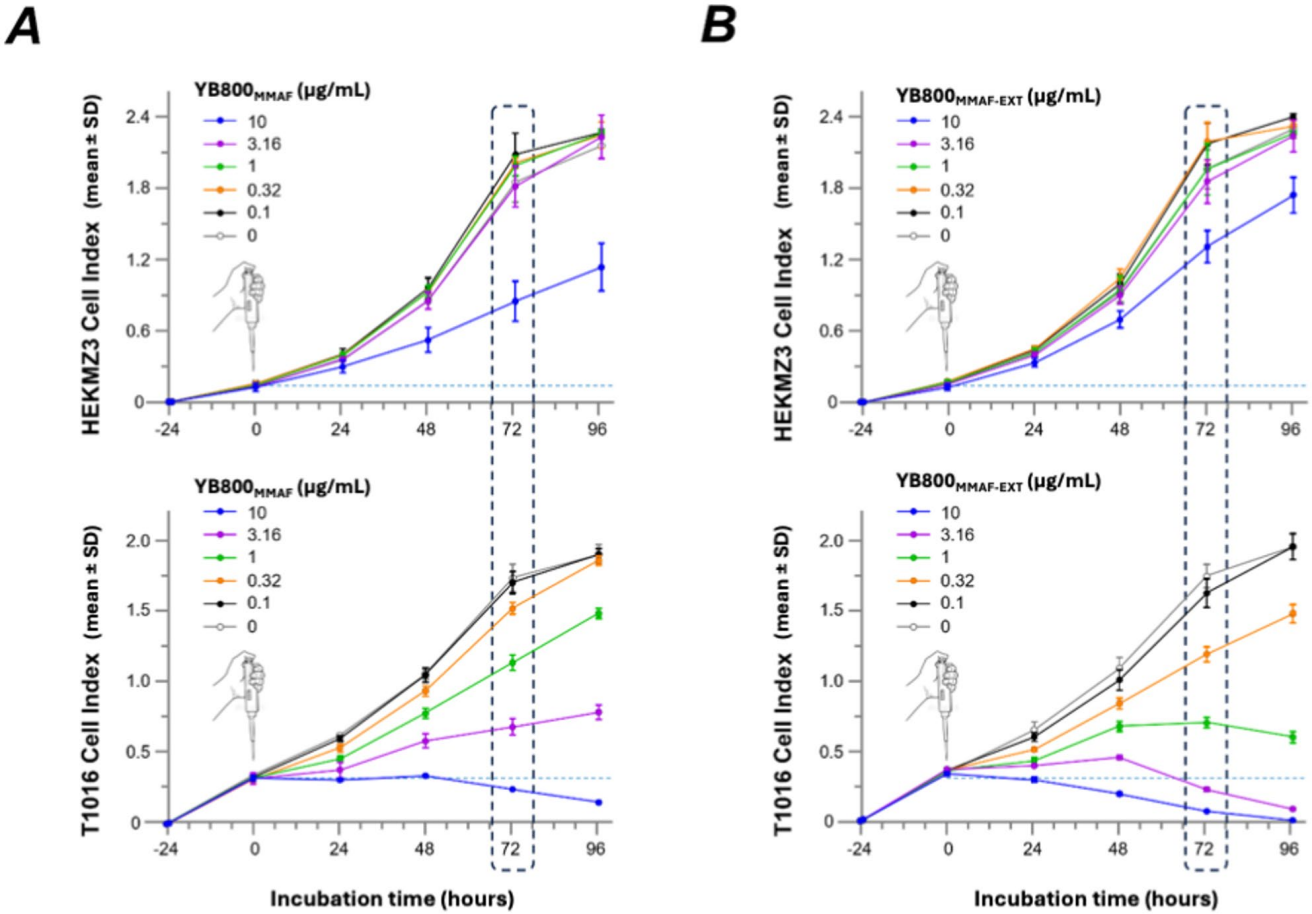
Time- and concentration-dependent effect of YB-800_MMAF_ or YB-800_MMAF−EXT_ on either control HEKMZ3 or T1016 cell proliferation/survival. HEKMZ3 (no *NPTXR* expressing cells) or T1016 engineered cells (transfected with human NPTXR-containing plasmid) were seeded on microtiter E-plates as indicated in the Methods section, and cells were allowed to adhere to plastic for 24 h at 37 °C in a humidified cell culture incubator in 5% CO_2_ atmosphere. Cells were then treated with the indicated concentrations (µg/mL) of either (**A**) YB-800_MMAF_ or (**B**) YB-800_MMAF−EXT_ and were monitored over a period of 96 h under similar cell culture conditions. Impedance was measured at various timepoints using the Agilent xCELLigence Real-Time Cell Analysis (RTCA) SP instrument, and reported as a cell index (CI) value. Specifically, the CI measurements occurred at cell seeding, 4 and 24 h after cell seeding (before compound addition), and at 24, 48, 72 and 96 h after compound addition. In this experiment, vehicle was sterile PBS. Data from 3 experimental replicates are shown

HEKMZ3 and T1016 cells were seeded onto 96-well E-plates (10,000 cells per well) and cultured in their respective culture media for 24 h at 37 °C in a humidified incubator with 5% CO_2_ atmosphere. Vehicle, cisplatin and either YB-800, YB-800_MMAF_, or YB-800_MMAF−EXT_ antibodies were then added at increasing concentrations before incubating for up to 96 h (4 days). Measurements were performed at cell seeding, 4, and 24 h (before test item addition), and at 24, 48, 72, and 96 h following test item addition (Figs. 1 and 2).

### Efficacy of YB-800_ADC_ in experimental subcutaneous established T1016 tumor model

Experiments were conducted at Rincon Bio (Salt Lake City, UT, USA). Five-week-old homozygous nu/nu immune-compromised female athymic nude mice (strain 002019) were procured through Jackson Laboratory (Bar Harbor, ME, USA) and were housed in Optimice carousel sterile quarters with filtered air supply in disposable cages from Animal Care Systems, Inc. (Centennial, CO, USA). Mice were fed ad libitum Teklad irradiated (sterilized) mouse diet and bedded with Teklad irradiated (sterilized) corncob bedding from Envigo RMS LLC (Indianapolis, IN, USA). T1016 cells were cultured as previously described and on the day of tumor cell implantation, cultured T1016 cells were trypsinized and allowed to detach from flasks. After trypsin neutralization and centrifugation, cells were resuspended in 50:50 Cultrex: RPMI (no supplementation); 100 µL (5 × 10^6^ cells) was injected subcutaneously into the right hind flank of 7-week-old mice. When average subcutaneous tumor volume reached approximately 90 mm^3^, mice were randomized to experimental groups (6–8 mice per group) and were treated intraperitoneally once a week (i.p., QW) for 3 to 4 weeks with PBS (vehicle) or 3–10 mg/kg/administration of either YB-800 or YB-800_MMAF−EXT_. Mice were monitored daily for behavior and food consumption, and twice a week for body weight and tumor volume (using the formula tumor volume = (L × W²)/2, via caliper-aided tumor size measurements) throughout the entire study. A consistent decrease in body weight greater than 20% and a tumor burden greater than 3,500 mm^3^ were commonly used as humane ethical endpoints for individual animal termination. Animals were monitored until Day 39–47 (i.e., end of the study), and Kaplan-Meier curves were generated. NPTXR protein expression in tumor samples was confirmed by IF, and cell proliferation was confirmed by IF using the cell proliferation marker Ki-67, with DAPI staining used to stain cell nuclei.

### Efficacy of YB-800_ADC_ in a subcutaneous established human bladder PDX tumor model

The human bladder PDX model (Blad15986) was established from tumor biopsy samples from a male Caucasian patient presenting with a urothelial carcinoma. The PDX model was amplified through serial passaging in severely immunocompromised NOG mice. Tumor fragments (approximately 10 mm^3^) from passage 7 were used for subcutaneous implantation (on Day 0) into the inguinal region of 20 g to 25 g female NOG mice (Taconic Biosciences, Denmark). Subcutaneous tumors were allowed to grow to 100–200 mm^3^ size, at which time (Day 49), tumor-bearing mice were randomized into treatment groups based on tumor size. Mice were then treated weekly for 3 weeks with intraperitoneal administrations of 100 µL of either vehicle (PBS) or 10 mg/kg/administration of YB-800_MMAF−EXT_. Animals were monitored daily for survival and clinical signs, while body weight and tumor size (using the formula tumor volume = (L × W²)/2, via caliper-aided tumor size measurements) were assessed twice weekly until decision to terminate the study. A consistent decrease in body weight greater than 20% or a tumor burden greater than 1,500 mm^3^ were commonly used as humane ethical endpoints for unplanned premature termination of individual animals.

## Results

### Expression of NPTXR in cancer and healthy tissues from humans

Semi-quantitative IF analysis of tissue microarrays demonstrated a high prevalence of membranous-cytoplasmic NPTXR expression in several human cancers, with the highest H-scores being reported in bladder cancer, cervical cancer, and non-small-cell lung cancer (Table 1).

**Table 1:** NPTXR expression in various cancers as evaluated by immunofluorescence

Cancer	*N*	Prevalence (%)	H-score^a^
Bladder	57	98.3	282.6
Cervix	39	89.7	220.4
Cholangiocarcinoma	54	62.9	140.6
Colon	45	97.8	177.9
Endometrium	39	71.8	161.5
Esophagus	60	61.6	123.2
Kidney	140	20.7	42.9
Lung	54	64.9	144.6
Lymphoma	24	0	0
Mammary (not triple negative)	71	93	201.5
Mammary (triple negative)	86	68.6	152.3
Neuroendocrine lung	39	18	33.5
NSCLC	50	82	210
Ovarian	47	57.4	127.5
Pancreas	48	79.2	202.1
Prostate	59	10.2	13.6
Rhabdomyosarcoma	192	0	0
Skeletal	44	0	0
Thyroid	46	2.2	4.4

^a^Total H-scores were calculated using the following formula: H-score = (1 × percentage of cells with low intensity staining) + (2 × percentage of cells with medium intensity staining) + (3 × percentage of cells with high intensity staining). NSCLC, non-small-cell lung cancer

**Fig. 3:**
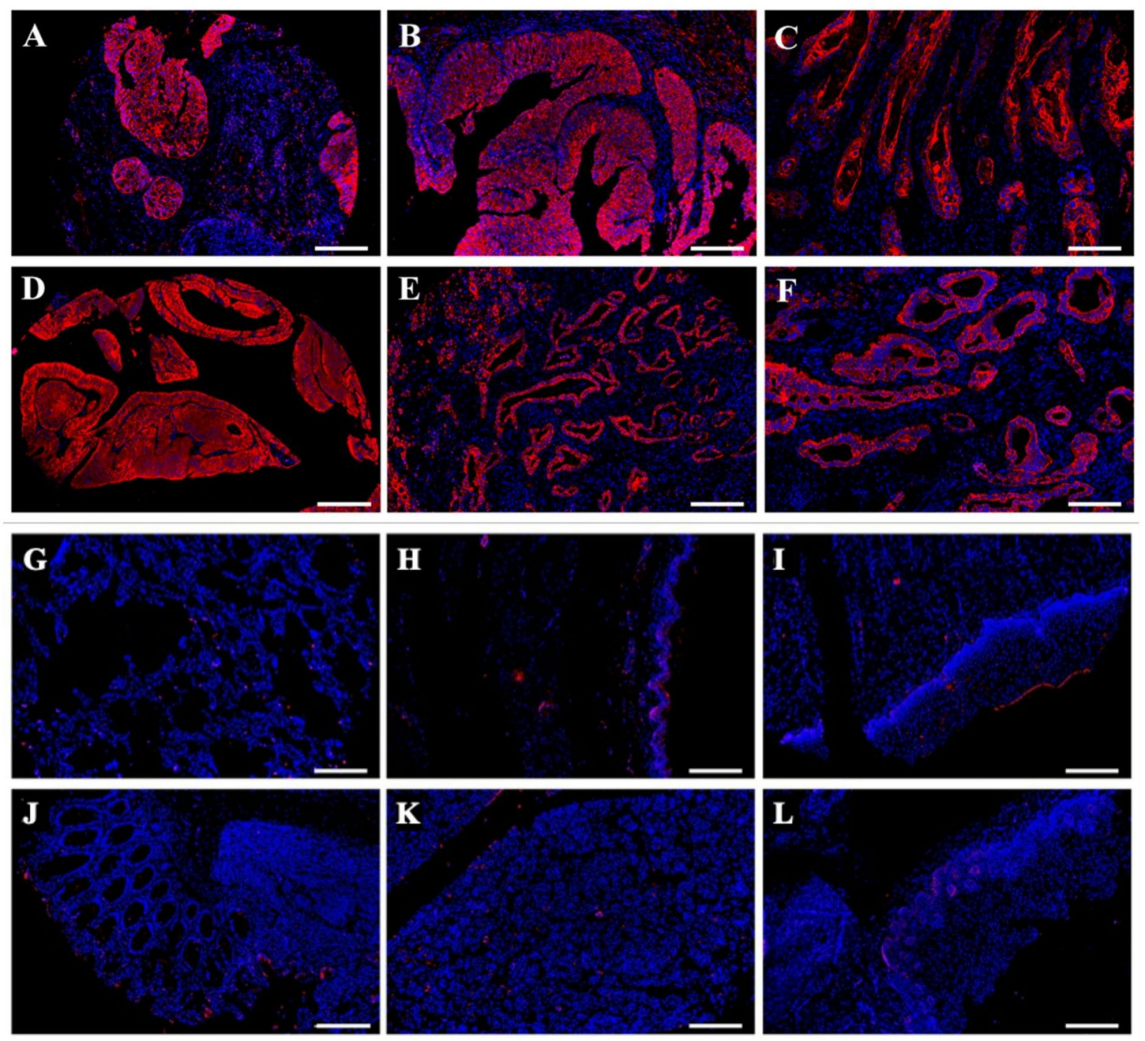
NPTXR expression in six representative tumors with medium to high H-scores and healthy tissues: (**A)** squamous non-small-cell lung cancer (T2N0M0, Stage Ib). (**B**) Invasive bladder cancer (T1N0M0, Stage I). (**C**) Adenocarcinoma of cervix, (T2bN0M0, Stage IIb). (**D**) Adenocarcinoma of colon (T3N1M0, Stage IIIb). (**E**) Pancreas carcinoma (T3N1aM0, grade 3, Stage IIb). (**F**) Adenocarcinoma of the esophagus (T3N1M0, grade 2, Stage IIIb). NPTXR expression in corresponding healthy tissue samples: (**G**) lung, (**H**) bladder, (**I**) cervix, (**J**) colon, (**K**) pancreas, and (**L**) esophagus. All scale bars 200 μm

NPTXR expression in six representative tumors with medium to high H-scores (squamous non-small-cell lung cancer, invasive bladder cancer, adenocarcinoma of the cervix, adenocarcinoma of the colon, pancreatic carcinoma, and adenocarcinoma of the esophagus) is shown in Fig. 3.

**Fig. 4:**
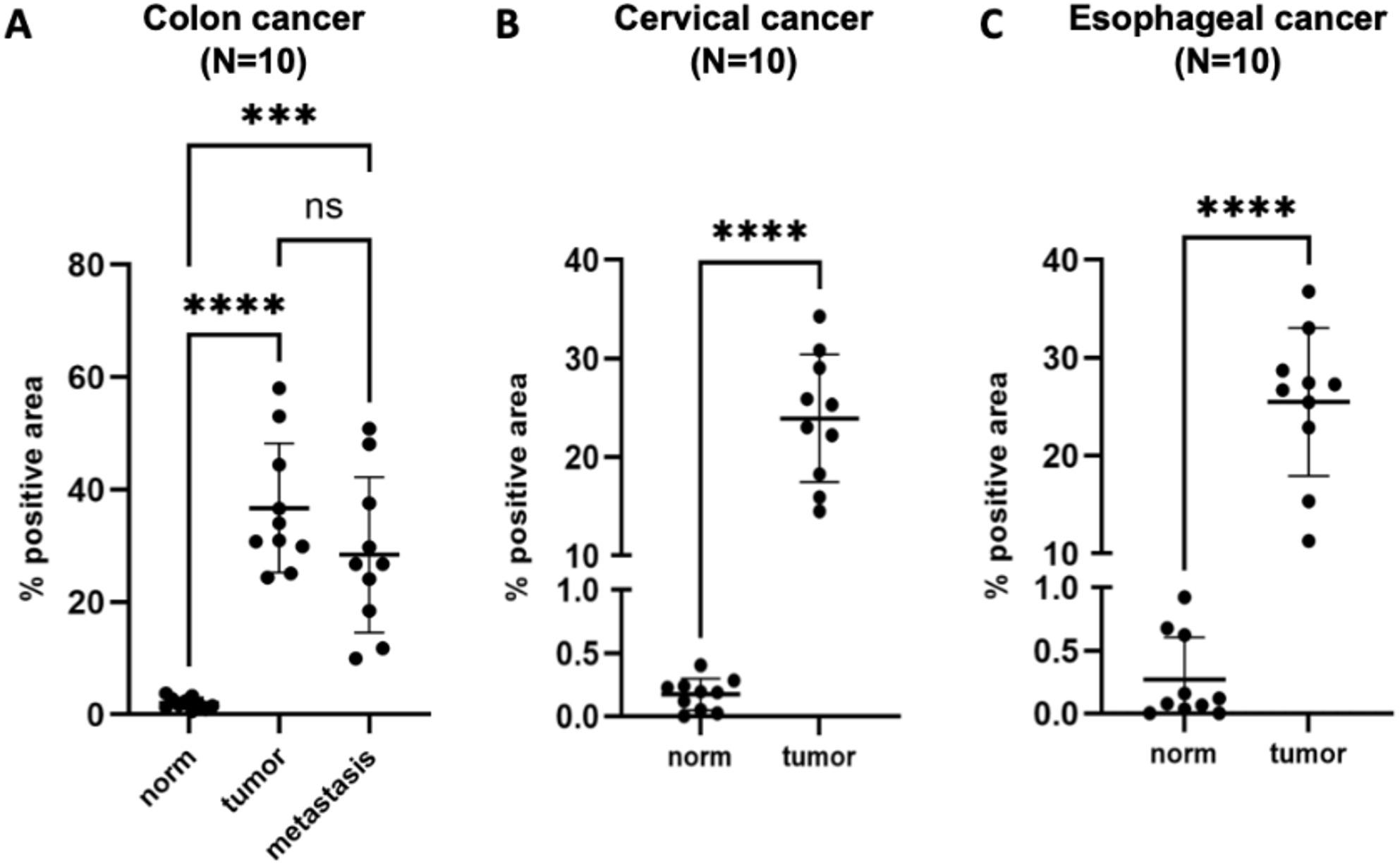
(**A**) Quantification of the Cy3-positive area (NPTXR signal) in normal tissue, tumor, and metastasis from different colon cancer patients (N = 10 images per group). (**B** and **C**) Quantification of the Cy3-positive area (NPTXR signal) in normal and tumor tissues from different cervical and esophageal cancer patients (N = 10 images per group). Comparisons between two groups (tumor vs. normal tissue) were performed using unpaired Student’s *t*-test. Comparisons among three groups (normal, tumor, and metastasis tissue across different patients) were carried out using one-way ANOVA followed by Bonferroni post hoc test. Statistical significance was defined as *p* < 0.001 (***) and *p* < 0.0001 (****)

NPTXR is highly expressed in tumor nests and non-tumor compartments (stroma, infiltrating cells) are negative, pointing to tumor-selective expression. In contrast, NPTXR expression in corresponding healthy tissue as shown in Fig. 3 show minimal and restricted straining, unlike in tumor tissue, and no large clusters of positive cells are present. Quantitative assessment of NPTXR expression in three representative tumors and healthy tissue show statistically significant and very strong differences in NPTXR staining between tumor tissues when compared with healthy tissue (Fig. 4).

Importantly, NPTXR expression is also highly prevalent in corresponding metastatic lesions of colon and pancreatic cancer (Table 2; Fig. 4; colon and pancreatic cancer metastasis are shown due to sufficient sample size).

**Table 2:** NPTXR expression in metastatic lesions of colon and pancreatic cancer as evaluated by immunofluorescence

Metastatic lesions (lung, liver, lymph node)	*N*	Prevalence (%)
Colon	8	100
Pancreas	14	79

### NPTXR expression in mice

When analyzed using murine tissue microarrays, NPTXR expression was observed in lung, cerebrum, and stomach tissue of mice; no NPTXR expression was observed in murine skeletal muscle, cardiac muscle, kidney, liver, or spleen (Supplementary Figure S7). Thus, in healthy mice NPTXR expression could be found in a limited number of additional organs compared to the expression of NPTXR in healthy human tissue.

### YB-800_ADCs_ activity in tumor cells expressing *NPTXR*

**Fig. 5:**
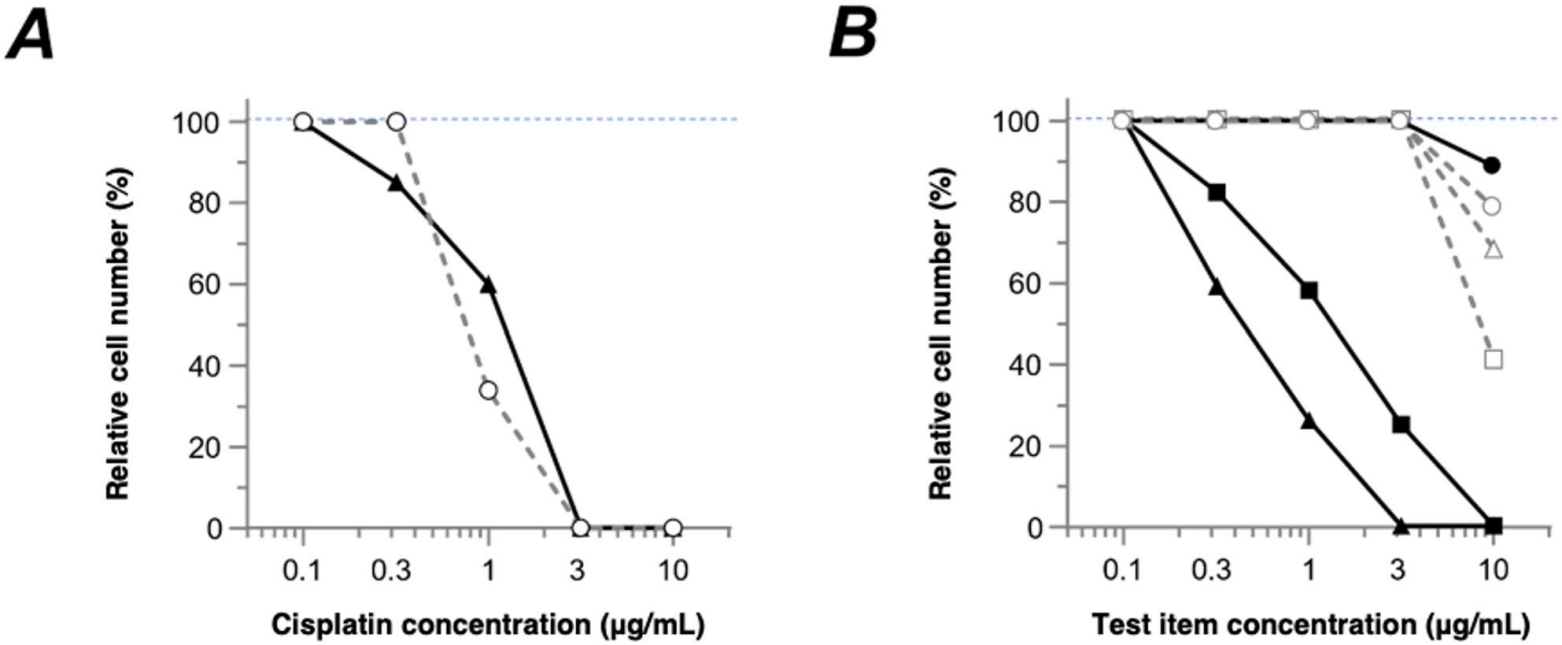
Concentration-dependent effect of cisplatin, YB-800 or YB-800_ADCs_ on either HEKMZ3 or T1016 cell proliferation/survival. HEKMZ3 (open symbols) or NPTXR-engineered T1016 (close symbols) cells were seeded on microtiter E-plates as indicated in the Methods section, and cells were allowed to adhere to plastic overnight at 37 °C in a humidified cell culture incubator in 5% CO_2_ atmosphere. Cells were then treated with the indicated concentrations (µg/mL) of either (**A**) cisplatin or (**B**) with YB-800 (open circle, black circle), YB-800_MMAF_ (open square, black square) or YB-800_MMAF−EXT_ (open triangle, black triangle), and were monitored over a period of 96 h under similar cell culture conditions. Impedance was measured at various timepoints using the Agilent xCELLigence Real-Time Cell Analysis (RTCA) SP instrument and reported as a cell index (CI) value as indicated in Figs. 1 and 2. Results represent the relative cell number (expressed as percentage) at 72 h – at which time cell culture remained exponential – based on CI in test item-treated versus vehicle-treated conditions. In this experiment, vehicles were sterile water or PBS for cisplatin and antibodies, respectively

Parental YB-800 and YB-800_ADCs_ were evaluated in T1016 and HEKMZ3 (control) cell-based assays for time- and concentration-dependent cell proliferation and survival using the xCELLigence technology platform (Figs. 1 and 2). These assays were specifically designed to evaluate target-dependent, antibody-mediated cytotoxicity in tumour cells overexpressing NPTXR. Importantly, target-negative HEKMZ3 cells were included as a negative control to demonstrate that the ADC-mediated cell killing is target-dependent and antibody-mediated. Validation with increasing concentrations of cisplatin (consider as an experimental positive control) showed similar concentration-dependent effect on both HEKMZ3 and T1016 cell proliferation and/or survival with IC_50_ estimated at approximately 1 µg/mL (Fig. 5A).

While parental YB-800 did not show any activity in either HEKMZ3 or T1016 cells, YB-800_ADCs_ showed a strong time- and concentration-dependent effect on T1016 cell proliferation/survival (with estimated IC_50_ of 0.5 and 2 µg/mL for YB-800_MMAF−EXT_ and YB-800_MMAF_, respectively), and limited, if any, activity in HEKMZ3 cells at relevant concentrations (Fig. 5B, and Figs. 1 and 2). Taken together, these results prompted consideration of YB-800_MMAF−EXT_ as the best candidate for further experimental evaluation.

### In vivo efficacy in mice

**Fig. 6:**
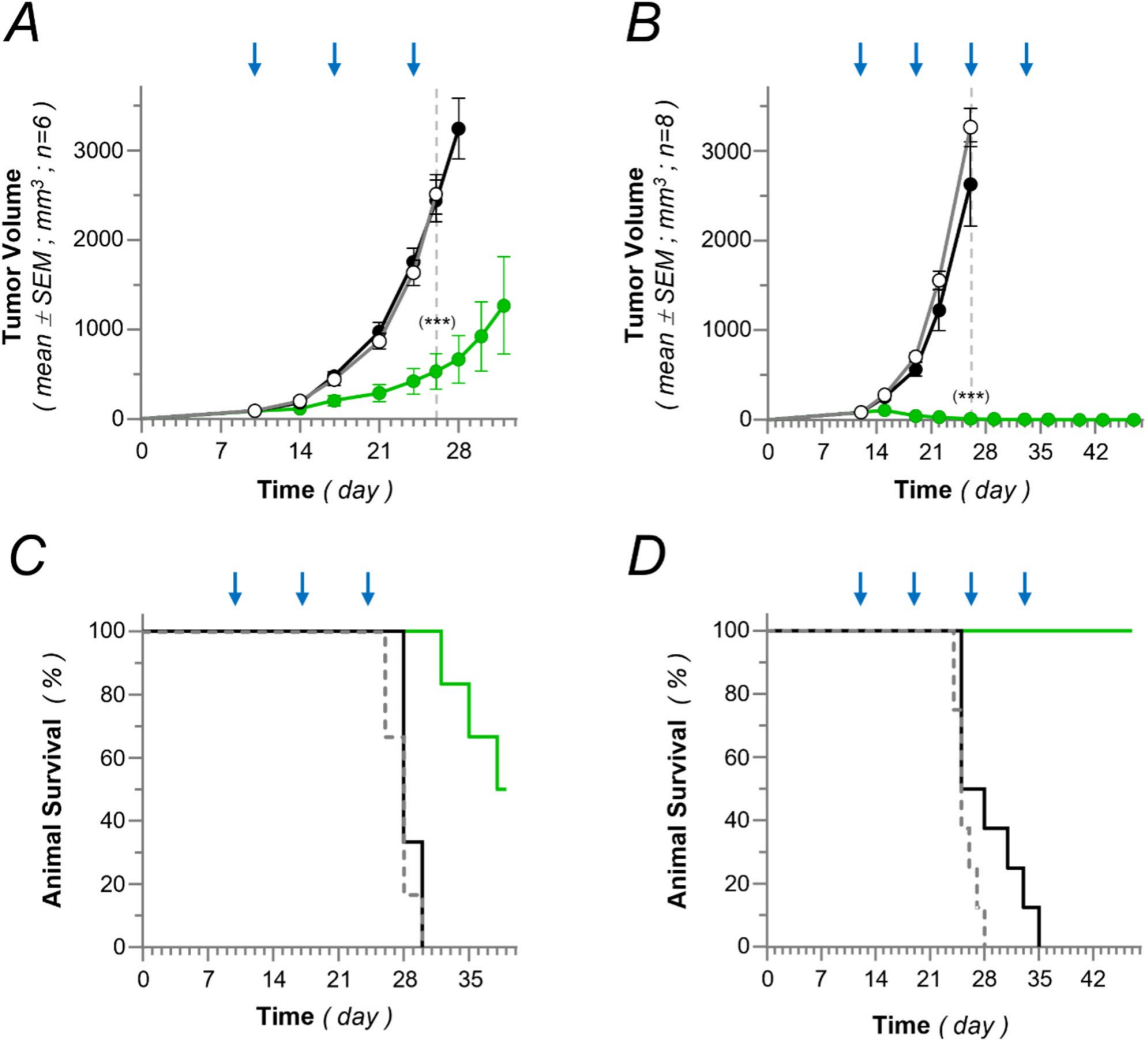
Time-dependent effect of YB-800 or YB-800_ADC_ on T1016 tumor growth. In 2 independent studies, T1016 cells (5 × 10^6^) were injected subcutaneously in the flank of female immuno-compromised BALB/c nude mice, and subcutaneous tumors were allowed to grow to approximately 100 mm^3^ mean tumor volume, at which time animals were randomized to the selected experimental groups (*n* = 6–8 animals per group). (**A**) Animals (*n* = 6 per group) were then treated systemically with either vehicle (PBS; open circle), or 3 mg/kg of YB-800 (black circle) or YB-800_MMAF−EXT_ (green circle) administered intraperitoneally weekly for 3 weeks. (**B**) Animals (*n* = 8 per group) were then treated systemically with either vehicle (PBS; open cicle), or 10 mg/kg of YB-800 (black circle) or YB-800_MMAF−EXT_ (green circle) administered intraperitoneally weekly for 4 weeks. In both studies, the time-dependent effect of the test items on T1016 tumor volume was monitored (measured in mm^3^), and results (expressed as mean tumor volume ± SEM) are depicted up to the time at which all animals were still present in each experimental group. At the time at which animal numbers were similar in all groups, statistical analysis - using GraphPad Prism 10 (GraphPad Software, San Diego, CA) - was performed using the two-tailed Mann-Whitney non-parametric test, as compared to vehicle control group (*** indicates *p* < 0.005). (**C**, **D**) Effect of PBS, YB-800 or YB-800_MMAF−EXT_ on individual T1016 tumor-bearing animal survival in the corresponding study (**A**, **B**, respectively) was monitored throughout the entire study till humane ethical animal termination or sponsor decision to terminate the study. Kaplan-Meier curves were generated for each experimental treated group: PBS (vehicle; dashed grey line), YB-800 (black line), and YB-800_MMAF−EXT_ (green line). Blue arrows indicate the timing of test item treatment

Immunofluorescence analysis of subcutaneous T1016 tumors (resulting from the injection of T1016 cells into the flank of immune-compromised mice) showed consistent expression of Ki67 protein (cell proliferation marker) throughout the tumor, demonstrating an actively proliferating experimental tumor model. In contrast, NPTXR positive staining was more heterogeneous, consistent with the polyclonal nature of T1016 cells. When established T1016 tumor-bearing animals were treated with either vehicle (PBS), or 3–10 mg/kg of either YB-800 or YB-800_MMAF−EXT_ administered systemically (i.p.) once a week for 3 to 4 weeks, experimental group analysis of the results (up to the time at which all animals were still alive in each group) clearly demonstrated a pronounced and statistically significant tumor growth inhibition as compared to vehicle control group upon treatment with YB-800_MMAF−EXT_, while no effect was observed with parental YB-800 antibody (Fig. 6A and B). The anti-tumor response to YB-800_MMAF−EXT_ was shown to be time- and dose-dependent as treatment with 3 mg/kg/administration led to a marked tumor growth inhibition (TGI, approximately 80%), while treatment at 10 mg/kg/administration led to complete tumor regression with treated animals showing no detectable tumors at necropsy. Interestingly, considering the observed heterogeneous expression of NPTXR in this tumor model, the pronounced anti-tumor activity observed with YB-800_MMAF−EXT_ may be correlated not only to the binding and direct effect of the ADC on tumor cells, but also to a potential efficient bystander (abscopal) effect on neighboring non-antibody-bound tumor cells. The presence of exatecan (a water-soluble, synthetic camptothecin analog that is able to cross the plasma membrane) in the dual payload ADC (YB-800_MMAF−EXT_) may explain this observed phenomenon (MMAF may not freely cross the plasma membrane).

Consistent with the observed tumor growth inhibition, treatment with YB-800_MMAF−EXT_ significantly prolonged animal survival (Fig. 6C and D), while parental YB-800 did not show any benefit to animal survival compared to the vehicle-treated group. Notably, animal survival was based only on an ethical tumor burden of 3,000 mm^3^ (at which volume animals were euthanized), and no unscheduled premature animal termination, macroscopic sign of tolerability/toxicity, nor gross organ examination findings at necropsy were observed throughout the study (data not shown).

**Fig. 7:**
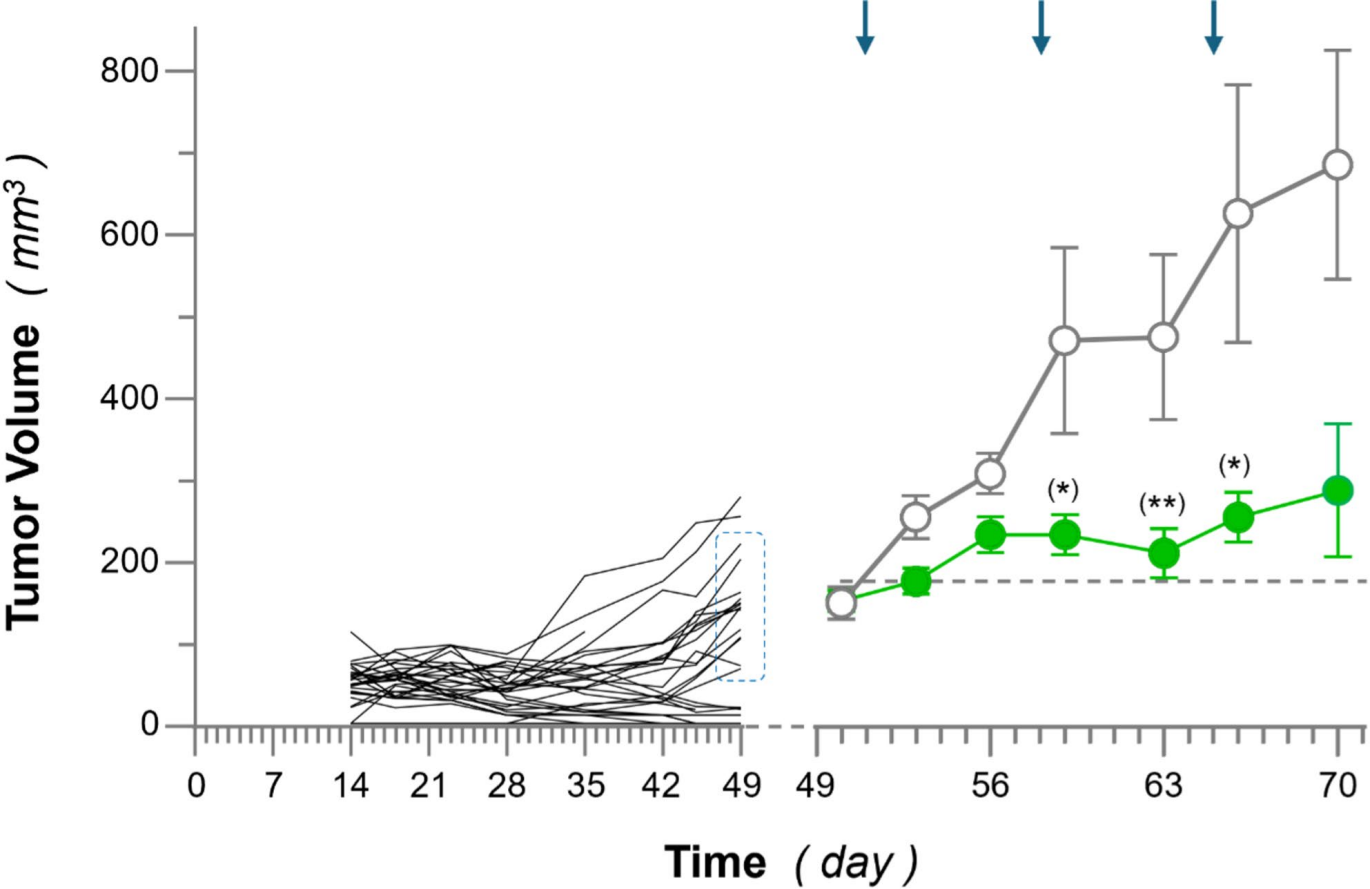
Time-dependent effect of vehicle or YB-800_MMAF−EXT_ on human bladder Blad15986 PDX tumor growth. Blad15986 tumor fragments (~ 50 mm^3^) were implanted subcutaneously in the flank of severely immuno-compromised NOG female mice (Day 0), and subcutaneous tumors were allowed to grow to 100–200 mm^3^ tumor volume (Day 49), at which time tumors were selected for consistent growth, as illustrated on the left part of the graph in which time-dependent individual tumor growth is depicted. Tumor-bearing animals were randomized to the selected experimental groups (*n* = 5–6 animals per group), and were then treated systemically with either vehicle (PBS; open circle), or 10 mg/kg of YB-800_MMAF−EXT_ (green circle) administered intraperitoneally weekly for 3 weeks. The time-dependent effect of test items on tumor volume was monitored (measured in mm^3^), and results (expressed as mean tumor volume ± SEM) are depicted up to the time (Day 70) at which all animals were still present in each experimental group (*n* = 4–5, as one animal in each group had to be terminated for human ethical endpoint). Statistical analysis - using GraphPad Prism 10 (GraphPad Software, San Diego, CA) - was performed using the two-tailed Mann-Whitney non-parametric test, as compared to vehicle control group (* indicates *p* < 0.05, and ** *p* < 0.01)

Regarding the evaluation of YB-800_MMAF−EXT_ in an experimental human bladder PDX (Blad15986) model, while animals in the control group exhibited time-dependent tumor growth, animals treated with YB-800_MMAF−EXT_ showed pronounced and statistically significant tumor growth inhibition (tumor stabilization) (Fig. 7). The study was prematurely terminated due to consistent body weight loss, which was observed equally in both groups. Under the given experimental conditions, all treatments were generally well tolerated, with only minor side effects observed. The human bladder Blad15986 PDX model demonstrated marked sensitivity to YB-800_MMAF−EXT_ treatment, achieving tumor stabilization TGI values of approximately 80% towards the end of the study.

## Discussion

Historically, the development of novel cancer treatments has been complicated by the need to kill cancerous cells while preserving surrounding healthy tissue. Whereas some cytotoxic drugs may be equally damaging to both the tumor and the surrounding tissue, monoclonal antibody-based therapies may not adequately destroy cancer cells despite being less toxic to healthy tissues. Antibody–drug conjugates combine cytotoxic drugs with highly specific monoclonal antibodies via chemical linkers to enable precise delivery of the cytotoxic agent to tumor cells [9–14]. Antibody–drug conjugates therefore represent a promising approach to cancer treatment as they allow cytotoxic agents to be specifically targeted to cancer cells, thereby maximizing the cytotoxic effects on tumors while sparing the surrounding healthy tissue [15]. As of 2025, the US FDA has approved 14 antibody–drug conjugates, with many more under development [16]. The efficacy of an antibody–drug conjugate is dependent on multiple factors: the monoclonal antibody should have high target specificity, high internalization efficiency, and a long half-life in circulation [16, 17], while the linker should remain stable in the circulation while also being efficiently cleaved in the target environment [16, 17]. Finally, the payload should have efficacy at very low concentrations. Tubulin polymerization inhibitors such as MMAF induce apoptosis through the blocking of microtubule polymerization; MMAF combined with a non-cleavable linker also has lower membrane permeability than the closely related molecule MMAE, meaning it is less likely to cross into adjacent, non-targeted and potentially healthy cells (the so-called ‘bystander effect) [18, 19]. Exatecan, which targets DNA replication and which can be paired with a cleavable linker, is cell permeable and usually provides better activity in heterogeneous tumors [20]. In this study, we first validated NPTXR as a new tumor marker and determined its expression in human cancer and healthy tissue. We first used IF analysis of human tissue microarrays to show that NPTXR is highly expressed on the cell membrane and cytoplasm in several human cancers, but shows only very limited, if any, expression in the majority of healthy tissue types. Expression of NPTXR was observed in the majority of all bladder cancer, colon cancer, squamous non-small-cell lung cancer, and breast cancer samples tested. Intertumoral expression of NPTXR was homogenous in more than 90% of cancer cells within each respective tumor sample. Our findings demonstrate that NPTXR has several key characteristics that make it an ideal tumor target. In particular, the high inter- and intra-tumoral homogenous expression in a number of solid tumors provides significant advantages compared with other tumor targets currently being explored or already approved in antibody–drug conjugate therapies for cancer. For example, our findings suggest that antibody–drug conjugate therapies that specifically target NPTXR may have favorable therapeutic indices, with only limited off-target effects on healthy tissue. *NPTXR* mRNA expression has previously been shown to be associated with disease progression in gastric cancer, and to function as an independent prognostic factor in this disease. Similarly, NPTXR protein expression has been suggested as a potential biomarker for disease recurrence in patients with gastric cancer [7]. Our findings are the first to suggest that NPTXR may function as a tumor marker for a wider array of solid tumor cancers, with potential applications for the early detection and diagnosis of a number of common cancers.

Having validated NPTXR as a tumor marker, we then designed, constructed, and evaluated antibody–drug conjugates based on YB-800, a first-in-class antibody that binds to NPTXR with high affinity. We demonstrated that the YB-800_ADCs_ (YB-800_MMAF_ and YB-800_MMAF−EXT_) inhibit tumor cell proliferation and induce tumor cell death in NPTXR-expressing HEK293 (T1016) cells in vitro. Finally, we also showed that YB-800_MMAF−EXT_ exerts pronounced inhibitory effects on tumor growth in immunocompromised mice bearing subcutaneous T1016 tumors or human bladder Blad15986 PDX. Mice were shown to have relevant NPTXR expression, and treatment with YB-800_MMAF−EXT_ was not associated with increased mortality or organ damage in mice.

Based on the design of YB-800_MMAF_ and YB-800_MMAF−EXT_, we expect that both antibody–drug conjugates exert their effects via intracellular release of cytotoxic agents in tumor cells upon internalization and transfer to the acidic lysosomal compartment, with extracellular vesicle-mediate transfer to adjacent tumor cells. In addition, exatecan contained in YB-800_MMAF−EXT_ is also expected to exert effects via direct release in the tumor microenvironment, with additional transfer of liposoluble cytotoxic agents to adjacent tumor cells. In conclusion, we have shown that the protein NPTXR is widely and selectively expressed in cancer cells, with only marginal expression in healthy tissues. This is a prerequisite for NPTXR to serve as a tumor marker for a variety of human cancers and as a new target for delivering cytotoxic therapy selectively to cancer cells. We have also developed a novel, first-in-class, fully humanized monoclonal antibody, YB-800, that targets human NPTXR and is efficiently internalized by cancer cells, which is required to show significant anti-tumor efficacy when conjugated with MMAF or MMAF and exatecan in the in vitro models. In addition, we have shown that the YB-800MMAF-EXT antibody–drug conjugate has a dose dependent anti-tumor effects in two in vivo models and may represent a promising new precision treatment for patients with solid tumors. Continued evaluation of YB-800_ADCs_ for the treatment of cancers is warranted.

## Electronic Supplementary Material

Supplementary Material 1

## Data Availability

The datasets used and analysed during the current study are available from the corresponding author on reasonable request.

## Abbreviations

AI: Artificial intelligence
CDR: Complementarity-determining region
CI: Cell index
DMEM: Dulbecco’s Modified Eagle Medium
EXT: Exatecan
FACS: Fluorescence-activated cell sorting
FBS: Fetal bovine serum
FC: Fragment crystallizable
IF: Immunofluorescence
IgG: Immunoglobulin G
IMDM: Iscove’s Modified Dulbecco’s Medium
IQR: Interquartile range
MFI: Mean fluorescence intensity
MMAF: Monomethyl auristatin F
MPA: Membrane protein array
NPTXR: Neuronal pentraxin receptor
PBS: Phosphate-buffered saline
ROI: Region of interest
SEM: Standard error of the mean
YB-800_ADCs_: Antibody–drug conjugates based on YB-800
