# Supplementary material for: NPTXR as a novel therapeutic target: efficacy of antibody-drug conjugates in preclinical tumor models
Source: Cancer Cell Int. 2026 Aug 7;26:278. doi: 10.1186/s12935-026-04438-5 (PMC13452120; doi:10.1186/s12935-026-04438-5)
Supplement: Supplementary file 1 [file 12935_2026_4438_MOESM1_ESM.docx]

# Supplementary Appendix

## Supplementary Methods

### Immunofluorescence staining and H-score assessment

Samples were deparaffinized with xylene 3 times for 10 minutes, before rehydrating in isopropanol, 96% ethanol, 70% ethanol, and distilled water (for 5 minutes each). Samples were then boiled in citrate buffer (pH 6.0) for 45 minutes at 97°C to 98°C and allowed to cool to room temperature for 15 to 20 minutes. Tissue sections were outlined with a PAP pen and washed in PBS for 5 minutes before permeabilization for 10 minutes in 0.1% Triton/PBS. Samples were washed again in PBS for 3 minutes before blocking for 60 minutes in 1% BSA/PBS and 5% IgG Goat. Primary antibody (anti-NPTXR diluted to 1:1000 in 1.0% BSA/PBS) was added overnight at 4°C, before washing in PBS twice for 10 minutes. Secondary antibody (biotinylated goat anti-human, Invitrogen, Thermo Fisher Scientific Cat. No. 31770, diluted 1:500 in 1.0% BSA/PBS) was added for 60 minutes, before again washing in PBS twice for 10 minutes. Finally, Cy3-streptavidin (Jackson ImmunoResearch, Cat. No. 016-160-084) was used to visualize biotinylated secondary antibodies bound to NPTXR, and DAPI (Serva, Cat. No. 18860) was used to stain cell nuclei. Both reagents were diluted 1:200 in 1.0% BSA/PBS and applied simultaneously as a single staining cocktail for 30 minutes, followed by two washes in PBS for 10 minutes each. Samples were washed in distilled water for 2 minutes, covered, and refrigerated for 30 minutes.

IF results were assessed semi-quantitatively by assessing the intensity of staining and the percentage of positively stained cells in the stained formalin-fixed paraffin-embedded slides.

Staining intensity was determined by a pathologist or scorer using either a fluorescence microscope or validated digital image viewer. Each section was assessed at a magnification of 4x to 10x and 3 to 5 representative non-overlapping regions were selected for scoring (avoiding edges, folds, necrosis, crush artifacts, and hemorrhages). Only viable invasive tumor cells were scored, with a minimum count of at least 500 cells across regions of interest.

Each region of interest was graded on a 0 to 3 scale, calibrated to positive controls (background was confirmed with a negative control). A staining grade of 0 indicated no detectable specific staining, 1 indicated weak staining that was barely above background, 2 indicated moderate staining, and 3 indicated strong staining with a robust or saturating signal.

The percentage of cells in each region of interest demonstrating each intensity grade was estimated to the nearest 5% (or to the nearest 1% if assessed digitally). Percentages were then averaged across regions of interest and H-scores were calculated using the following formula: H-score = (1 × percentage of cells with low-intensity staining) + (2 × percentage of cells with medium-intensity staining) + (3 × percentage of cells with high-intensity staining). H-scores ranged from 0 to 300 (low intensity: 0–100, medium intensity: 101–200; high intensity: 201–300), and, based on prior pre-clinical studies involving antibody–drug conjugates, an H-score threshold of ≥ 150 was considered to represent sufficient target expression to allow for sufficient antibody–drug conjugate binding and internalization to achieve therapeutic efficacy.

| **Antibody** | **Antigen** | **EC50, ng/mL  (95% confidence interval)** | **R^2^** | **Yield, mg/L** | **Total germline identity** | **Germline identity without CDR3** | **Germline identity  framework** |
| --- | --- | --- | --- | --- | --- | --- | --- |
| Anti-NPTXR_VH_ parental_VK_ parental | Strep + rhNPTXR-bio | 19.74 (16.25, 23.94) | 0.9941 | 32 | 71.25 | 73.35 | 73.65 |
|  | Strep + Bio-mNPTXR | 43.23 (35.19, 52.93) | 0.9923 |  |  |  |  |
| Anti-NPTXR_VH_ parental_VK3 | Strep + rhNPTXR-bio | 33.13 (29.59, 39.32) | 0.9981 | 17 | 75.45 | 77.9 | 80.95 |
|  | Strep + Bio-mNPTXR | 65.48 (57.45, 74.53) | 0.997 |  |  |  |  |
| Anti-NPTXR_VH1_ VK_parental | Strep + rhNPTXR-bio | 36.18 (33.29, 39.32) | 0.9989 | 53 | 78.35 | 81.2 | 86 |
|  | Strep + Bio-mNPTXR | 34.92 (29.75, 40.89) | 0.996 |  |  |  |  |
| Anti-NPTXR_VH1_ VK1 | Strep + rhNPTXR-bio | 141.1 (133.0, 149.8) | 0.9994 | 33 | 87.7 | 91.3 | 100 |
|  | Strep + Bio-mNPTXR | 157.7 (147.2, 169.0) | 0.9992 |  |  |  |  |
| Anti-NPTXR_VH1_ VK1*_Nagoya | Strep + rhNPTXR-bio | 68.37 (58.48, 79.75) | 0.996 | 37 | 89.1 | 92.8 | 99.4 |
|  | Strep + Bio-mNPTXR | 76.64 (67.54, 86.90) | 0.9973 |  |  |  |  |
| Anti-NPTXR_VH1_ VK3 | Strep + rhNPTXR-bio | 63.81 (57.16, 71.17) | 0.9981 | 36 | 82.55 | 85.75 | 93.3 |
|  | Strep + Bio-mNPTXR | 71.87 (64.85, 79.62) | 0.9983 |  |  |  |  |
| Anti-NPTXR_VH2_ VK_parental | Strep + rhNPTXR-bio | 43.20 (40.85, 45.68) | 0.9996 | 41 | 73.9 | 76.15 | 79.25 |
|  | Strep + Bio-mNPTXR | 31.14 (28.31, 34.24) | 0.9986 |  |  |  |  |
| Anti-NPTXR_VH2_ VK1 | Strep + rhNPTXR-bio | 83.22 (78.81, 87.88) | 0.9995 | 23 | 83.25 | 86.25 | 93.25 |
|  | Strep + Bio-mNPTXR | 67.19 (61.62, 73.25) | 0.9988 |  |  |  |  |
| Anti-NPTXR_VH2_ VK1*-Nagoya | Strep + rhNPTXR-bio | 57.94 (54.53, 61.56) | 0.9995 | 27 | 84.65 | 87.75 | 92.65 |
|  | Strep + Bio-mNPTXR | 43.69 (38.70, 49.28) | 0.9977 |  |  |  |  |
| Anti-NPTXR_VH2 _VK3 | Strep + rhNPTXR-bio | 38.89 (35.59, 42.47) | 0.9988 | 28 | 78.1 | 80.7 | 86.55 |
|  | Strep + Bio-mNPTXR | 39.86 (35.93, 44.19) | 0.9983 |  |  |  |  |

**Supplementary Table S1.** Characterization of antibody candidates. (**A**) Degree of humanness as calculated by germline identity (GI), production yield, and binding to NPTXR (ELISA).

**Supplementary Figure S1.** Characterization of antibody candidates. (**A**) Receptor inhibition testing data for antibody candidates. (**B**) Polyreactivity testing data for antibody candidates.

**Supplementary Figure S2.** Further characterization of YB-800. (**A**) Biolayer interferometry trace showing loading, association, and dissociation for humanized YB-800. (**B**) Global fitting for three concentrations of YB-800 (15.8 nM, 1.58 nM, and 5 nM) and negative control (palivizumab). (**C**) Antibody heat stability data.

**Supplementary Figure S3.** Structures of the YB-800-based antibody–drug conjugates YB-800_MMAF_ and YB-800_MMAF-EXT_. EXT, exatecan; MMAF, monomethyl auristatin F.

**Supplementary Figure S4.** Affinity measurement data for YB-800, YB-800_MMAF_, and YB-800_MMAF-EXT_.

**Supplementary Figure S5.** Further characterization of YB-800_MMAF_ and YB-800_MMAF-EXT_. (**A**) Drug-to-antibody ratio (DAR) analysis by matrix-assisted laser desorption/ionization time-of-flight mass spectrometry (MALDI-TOF-MS). For YB-800_MMAF_, based on molecular weight calculation, peaks *(A)* and *(B)* were identified as the singly and doubly charged YB-800_MMAF_, which carries four 2 MMAF drug bundles with a DAR value of 8. The predicted molecular weight of YB-800 conjugated with four MMAF drug bundles was 159,797 Da.

The difference between the predicted (148,717 + 4 × 2,770 = 159,797 Da) and

measured (159,867 Da) molecular weight was less than the system error (0.3%). These results demonstrate that the ADC carries four MMAF drug bundles, comprising a total of eight MMAF molecules. For YB-800_MMAF-EXT_, based on the molecular weight calculation, peaks *(C)* and *(D)* were assigned to the singly and doubly charged forms of YB-800_MMAF-EXT_, respectively; YB-800_MMAF-EXT_ was determined to carry four MMAF-EXT drug bundles, corresponding to a DAR of 8. The predicted molecular weight of YB-800 conjugated with four MMAF-Ext drug bundles was 161,899 Da. The difference between the predicted molecular weight (148,707 + 4 × 3,298 = 161,899 Da) and the measured molecular weight (162,031 Da) was less than system error (0.3%). These results demonstrate that the ADC carries four MMAF-EXT drug bundles, comprising a total of four MMAF molecules and four exatecan (EXT) molecules. (**B**) Purity analysis by size-exclusion high-performance liquid chromatography with ultraviolet detection (SEC-HPLC-UV). The percentage of high molecular weight in YB-800_MMAF_ sample was 2.00%, and an impurity was observed at 7.636 min at 4.83%. The percentage of high molecular weight in YB-800_MMAF-EXT_ sample was 3.42%. (**C**) Purity analysis by hydrophobic interaction chromatography-high performance liquid chromatography with ultraviolet detection (HIC-HPLC-UV) of the anti-NPTXR-azido (top image), YB-800_MMAF_ (left), and YB-800_MMAF-EXT_ (right). The purity of anti-NPTXR-azido was 100%. The conjugation purity of YB-800_MMAF_ was 96.59% (relative retention time versus anti-NPTXR-azido was 1.21), and the conjugation purity of YB-800_MMAF-EXT_ was 100% (relative retention time versus anti-NPTXR-azido was 1.19).

**Supplementary Figure S6.** Generation of human NPTXR-expressing HEK293 cells. The SV40 large T antigen-transformed human embryonic kidney HEK293T cells were transfected with the human NPTXR coding region-containing plasmids in Inscreenex proprietary *BXB1* or *Flp* landing pad (**E)**. Briefly, these plasmids contain a recombination site and a selection marker (blasticidine and G418 for *BXB1* and *Flp*, respectively) at a pre-validated genomic locus. Using a matching recombinase specifically designed expression setups, the target DNA is inserted into that locus, allowing for reproducible integration at well-defined sites in the genome. Polyclonal transfected cells were cultured in RPMI 1640 (containing L-Glu) cell culture medium complemented with 10 mM Hepes buffer pH 7.4, 10% fetal bovine serum, and corresponding selection marker (5 µg/mL and 1 mg/mL for blasticidine and G418, respectively). Expression of the target was then analyzed by flow cytometry using YB-800 as the target-specific antibody in control HEKMZ3 (HEK293 cells transfected without NPTXR-containing plasmids) and NPTXR-transfected cells, and either rat or goat anti-human IgG Fc fluorescently labeled antibodies at 1:100 dilution (Rat PE, Biolegend Cat.#410708; Goat AF488, ThermoFisher, Cat.#A-11013). FACS profiles were obtained for (**A**) T1000, (**B**) T1017, (**C**) T1005, and (**D**) T1016 polyclonal cells; grey lines represent control HEKMZ3 cells, and blue lines/areas represent the NPTXR gene-transfected cells. (**E**) Based on the extent of NPTXR cell membrane protein expression (mean fluorescence intensity) and sharpness of the profile, stably transfected polyclonal T1016 cells were selected as NPTXR-expressing cells for experimental proof of concept.
